# Mobile Health–Based Motivational Interviewing to Promote SARS-CoV-2 Vaccination in Rural Adults: Protocol for a Pilot Randomized Controlled Trial

**DOI:** 10.2196/64010

**Published:** 2025-04-28

**Authors:** Ashlea Braun, Sarah Corcoran, Khue Tu Doan, Cameron Jernigan, Cate Moriasi, Michael Businelle, Thanh Bui

**Affiliations:** 1 Health Promotion Sciences Hudson College of Public Health University of Oklahoma Health Sciences Tulsa, OK United States; 2 Department of Nutritional Sciences Oklahoma State University Stillwater, OK United States; 3 TSET Health Promotion Research Center Stephenson Cancer Center University of Oklahoma Health Sciences Oklahoma City, OK United States; 4 Hudson College of Public Health University of Oklahoma Health Sciences Oklahoma City, OK United States; 5 Department of Family and Preventive Medicine College of Medicine University of Oklahoma Health Sciences Oklahoma City, OK United States

**Keywords:** motivational interviewing, vaccination hesitancy, community-based participatory research, rural health, mobile health, mHealth

## Abstract

**Background:**

Despite documented effectiveness, the public health impact of vaccinations is severely limited by misperceptions, hesitancy, and poor acceptance. Messaging from health care providers has not yet been optimized to overcome these barriers and has not been tailored to groups that face health disparities, such as rural Americans. Because vaccines have become controversial, as illustrated by the public response to the SARS-CoV-2 vaccines, traditional approaches that use persuasive education or advice to change perspectives are unlikely to have long-term effects and may even be counterproductive. Alternatively, motivational interviewing (MI) is a conversational approach to address modifiable behavior and its empathic nature can be useful when navigating challenging topics. Although MI has been found to be efficacious in improving vaccination rates among children and adolescents, it is unknown whether MI can reduce vaccine hesitancy and health disparities among underserved rural adults. Further, the ideal mode of delivery for MI is unknown, especially “dose,” “intensity,” and integration with mobile health (mHealth). Therefore, it is essential to investigate the efficacy of MI in promoting vaccine uptake in rural populations to reduce health disparities.

**Objective:**

This study aims to develop and evaluate the feasibility, acceptability, and preliminary efficacy of our mHealth-based MI intervention to diminish SARS-CoV-2 vaccine hesitancy (MOTIVACC).

**Methods:**

This pilot study uses mixed methods. A 2-phase study will be conducted: convening a community advisory panel to understand barriers and facilitators to vaccination and mHealth uptake among adults (phase 1, n=16-20), and a pilot 3-group single-blind randomized controlled trial (RCT) for 8 weeks (phase 2, N=60). In the RCT, we recruit adults who have received no previous dose of the COVID-19 vaccine and randomize them into one of three arms: standard MI (SMI; n=20), intensive MI (IMI; n=20), or mHealth-based MOTIVACC (n=20). The primary RCT outcomes are positive change in vaccine hesitancy and intention to obtain the vaccines, measured on Likert scales. The secondary RCT outcome is the actual vaccine receipt.

**Results:**

Phase 1 of this study was approved by the ethics committees of both the University of Oklahoma and Oklahoma State University in July 2022, and was completed in June 2023. Phase 2 of this study was approved by the ethics committee at the University of Oklahoma in April 2024.

**Conclusions:**

This randomized trial will evaluate the preliminary efficacy of MI for targeting SARS-CoV-2 vaccine hesitancy, as well as compare traditional MI versus mHealth-based MI. This will provide pivotal data on scalable strategies to assist in navigating vaccine hesitancy, including in rural populations.

**Trial Registration:**

ClinicalTrials.gov NCT05977192; https://clinicaltrials.gov/study/NCT05977192

**International Registered Report Identifier (IRRID):**

DERR1-10.2196/64010

## Introduction

Throughout the COVID-19 pandemic, rural populations have had lower rates of SARS-CoV-2 vaccination, increased vaccine hesitancy, and higher risks of morbidity and mortality associated with active infection than urban populations [1]. Increased likelihood of vaccine hesitancy has been associated with rural residence, low household income, and less education [2]. While some strides have been made in rural vaccination, it has been estimated that those who remain unvaccinated and currently reside in rural areas are less likely to consider vaccination (eg, will “definitely not” get vaccinated) [3]. In Oklahoma specifically, as of May 10, 2023, 68% of Oklahomans aged 18–35 years and 57% of Oklahomans aged 36-49 years had not received any dose of a SARS-CoV-2 vaccine [4]. However, in urban counties, 76%-95% of adults had received at least one dose, while the rates in rural counties ranged from 37%-65% [5]. Further, it has been estimated that as many as two-fifths of rural Oklahoman residents are hesitant to receive the vaccine [6].

Health communication has been consistently identified as a key element in promoting vaccine uptake and addressing vaccine hesitancy. Historically, approaches taken to communication vary [7] but may focus on attempts to craft persuasive informational messages [8]. The effect of these messages is likely minimized, as many individuals who are vaccine-hesitant perceive the source of information (eg, health care providers) as misinformed [8], thus a fundamental shift in the purpose of vaccine-related communication is essential. Such approaches may instead focus on honoring individual autonomy and navigating information and perceptions of information, versus relaying of information itself [9]. Data also suggest the advantage of using messages that emphasize the personal benefits of vaccination rather than prosocial benefits in reducing vaccine hesitancy; however, this may differ according to individual values [10]. Thus, it is critical to align individual priorities with intervention content, a key feature of motivational interviewing (MI) [11]. No studies have examined the effects of MI on vaccination rates among adults most vaccine-hesitant. MI has documented efficacy in vaccine-hesitant parents (eg, child HPV vaccines): vaccine intention rose by 12%, hesitancy decreased by 40% [12], and vaccine coverage in infants increased by 3.2%, 4.9%, and 7.3% at 3, 5, and 7 months of age, respectively [13]. MI has also been used to promote HPV vaccination in high-risk adults with promising results [14], but not necessarily in those who are vaccine-hesitant. While promising, MI delivery can be laborious, limiting scalability. Alternatively, mobile health (mHealth)–based interventions have been used to promote vaccination [15]. Thus, integrating MI into automated platforms is essential for scalability to improve public health.

The World Health Organization (WHO) acknowledges mHealth as a cost-effective, scalable, and sustainable way to improve public health [16]. A limiting factor in MI’s effectiveness is scalability due to the intensity of provider training and evaluation. Further, smartphone ownership in the United States increased from 35% in 2011 to 90% in 2023 [17] with similar rates across urban and rural groups (91% and 87%, respectively). Near-universal smartphone ownership provides an ideal yet untapped mechanism to deliver MI-based vaccine interventions to rural populations.

In summary, there is a critical need for improving SARS-CoV-2 vaccination rates among rural populations in Oklahoma and nationwide. To address this need, we will test the efficacy of an mHealth-based MI intervention to diminish SARS-CoV-2 vaccine hesitancy (MOTIVACC). This protocol paper aims to describe our mixed-methods approach to develop and pilot test the MOTIVACC. This study and subsequent course of research will contribute significantly to reducing vaccine-preventable morbidity and mortality in rural populations.

## Methods

### Study Design

This pilot study uses a mixed-methods, empathy-driven, user-centered intervention design strategy with two phases: (1) convening a community advisory panel (CAP) to understand emerging and current barriers and facilitators to vaccination and mHealth uptake among rural adults and (2) a pilot 3-group, 8-week-long randomized controlled trial (RCT) to examine the feasibility, acceptability, and preliminary efficacy of MOTIVACC. The CONSORT (Consolidated Standards of Reporting Trials) and SPIRIT (Standard Protocol Items: Recommendations for Interventional Trials) checklists were used.

### Conceptual Framework

Our study (particularly qualitative data analysis, MI interventions, and RCT measures) is guided by social cognitive theory (SCT), which posits that an individual’s cognition and health behavior change in a social context with dynamic and reciprocal interactions with other people and the environment [18]. Key constructs of SCT include expectations (eg, vaccination benefits or consequences of not being vaccinated), observational learning (eg, seeing the vaccine’s effectiveness), self-efficacy (eg, vaccine confidence), reciprocal determinism (eg, the dynamic and reciprocal interactions of the person with other people and the environment, that leads to vaccine acceptance, including eliminating stigma), reinforcements (ie, internal or external processes to vaccine acceptance or uptake), and behavioral capability (ie, the ability to obtain the vaccine through sufficient knowledge and skills) [19]. Interactions within the MI intervention will help to address reciprocal determinism and reinforcements, as it will entail direct interactions with personnel to address ambivalence toward vaccination, identify discrepancies between vaccination decisions and individual values, and provide information to participants in an empathetic and value-driven manner [11,19].

### Phase 1: Intervention Development

#### Panel Development

We convene the rural CAP using the “broad community” model to capture participants’ opinions about vaccination [20]. We work directly with Cooperative Extension Educators at Oklahoma State University (OSU) to identify and partner with (1) key stakeholders (eg, community leaders) and (2) members of the community for panel development. Cooperative Extension is a key branch of the land-grant system and includes community outreach and health-related engagement with populations across the state, including rural adults [21]. We use purposive sampling to identify, with educators’ input, and recruit key partners within rural counties with the lowest vaccination rates at the time of recruitment. We recruit a minimum of two individuals in the bottom 10% of vaccinated counties across Oklahoma to participate in the CAP. Our target total sample size for phase 1 is 15-20 CAP participants in order to achieve this benchmark, which is consistent with existing recommendations in qualitative research [22].

#### Eligibility Criteria

Inclusion criteria include being ≥18 years of age, residing in a target county and being identified as a key stakeholder, and willingness to provide informed consent and complete a semistructured interview. There are no exclusion criteria.

#### Interview Procedure

We conduct one-on-one semistructured interviews with each participant (see Multimedia Appendix 1). Interviews include open-ended questions to gauge barriers and facilitators to vaccination. To promote the diversity of opinions, research personnel are trained in using open-ended questions that are developed with empathy-driven, user-centered principles [23] and in using MI-consistent prompts (eg, “Please tell us about the impressions of vaccines in your community” or “Please share your experience with vaccinations”). For individuals who express vaccine resistance, questions focus on their perspectives (eg, “Tell me what you don’t like about vaccines”) and hypotheticals (eg, “Imagine you were to get the vaccine, what would those circumstances look like?”). For those who have been vaccinated, questions similarly focus on community perceptions and participants’ own experiences (eg, “Tell me about your decision to get the vaccine”). Lastly, questions also center on mHealth (eg, “Imagine you were to use an app to help you make health care decisions – what would that look like to you?”). All interviews are about 30-45 minutes via phone, video call, or in-person if preferred. Interviews are recorded and transcribed verbatim for analysis.

#### Qualitative Data Analysis

Interviews conducted with the CAP are transcribed verbatim and triple-coded by 3 separate members of the research team to inductively identify themes classified broadly as barriers to and facilitators of receiving the COVID-19 vaccine as well as the use of mHealth using content analysis. In addition, all transcripts are reviewed for identification of SCT constructs and opportunities for the use of MI relational skills, technical skills, and existing empirical evidence [24,25]. This approach uses a reflexive strategy to integrate three different researchers’ perspectives into the data set (eg, experience in SCT versus MI) [26]. Coding matrices are used to match MI- and SCT-specific elements to barriers to and facilitators of vaccination to design potential mHealth-specific messages. Discrepancies in coding are discussed to achieve intercoder agreement, and inductive thematic saturation is achieved at the time of analysis by iteratively reviewing the emergence of new themes during triple coding [27]. If saturation is not achieved in that new themes emerge at the time of analysis that are not well integrated into the codebook, additional community members will be recruited to complete interviews (maximum total n=20) [28]. The final data set is entered into the NVivo software (Lumivero, LLC) package [29]. Codes are organized into barriers and facilitators; other categories can be created as they emerge during the coding process.

### Phase 2: Pilot RCT

#### Participant Recruitment

We leverage multiple recruitment strategies in an intentional effort to reach historically underrepresented areas across Oklahoma. For phase 2, we partner with OSU Cooperative Extension and TrialFacts to target primarily rural counties to recruit a total of 60 participants. Around 60 participants are selected as consistent with recommendations for pilot studies, taking into consideration the focus on evaluating feasibility, as well as practical restraints [30]. TrialFacts is a participant recruitment service company that provides targeted recruitment of specific populations of interest.

#### Eligibility Criteria

Eligible adults are (1) aged ≥18 years, (2) living in a rural area (based on Rural-Urban Commuting Area codes; rural, codes 4-10) [31,32], (3) self-report of never having received any dose of a SARS-CoV-2 vaccine, (4) able to speak English, and (5) able to provide written informed consent. Exclusion criteria include (1) a cognitive or other disability that inhibits smartphone use, (2) inability to participate because of medical or psychiatric conditions diagnosed by a physician or clinician, or (3) enrollment in other COVID-19 research.

#### Procedure

RCT participants (N=60) are randomized to one of the 3 treatment groups using simple randomization in the REDCap (Research Electronic Data Capture; Vanderbilt University) program [33,34]. These groups include standard MI (SMI; n=20), intensive MI (IMI; n=20), or mHealth-based MOTIVACC (n=20). SMI consists of one MI session at least 20 minutes in length [35-38] delivered by a trained counselor at the baseline and provision of printed self-help materials. IMI consists of the SMI component plus 3 additional MI sessions delivered by a trained counselor via telephone or video calls at weeks 2, 4, and 6 post enrollment. MOTIVACC consists of an empathy-driven, fully automated, smartphone-based MI program that involves proactive, interactive, and individually tailored messages, plus some embedded images or videos, delivered by our Insight app [39]. MOTIVACC will begin immediately after enrollment and data collection and continue for an 8-week period (about 2-5 messages or questions per day, depending on participants’ interaction and responses). The intervention content in all groups is adapted from the team’s previous work and existing empirical evidence for in-person MI [25], is informed by Phase 1 outcomes, and is designed to tap SCT mechanisms. Insight for Android is used and any participants enrolled in MOTIVACC who do not have an Android phone will be loaned one for the duration of the study. All participants are followed up at week 8 post enrollment via individualized REDCap survey links sent via email [33,34].

#### Outcomes and Measures

Primary assessments (self-reported data) are conducted at baseline (after completing informed consent) and 8 weeks (after completion of the intervention if applicable). Additional data regarding the completion of the intervention contacts is recorded at the time of delivery in REDCap by study personnel (ie, for MI) or consistently and automatically in Insight (ie, for MOTIVACC). Table 1 displays the assessments and measures. The primary RCT outcomes are positive changes in the vaccine hesitancy scale [40,41] and intention to obtain the vaccine [42,43]. The secondary outcome is actual vaccination.

**Table 1:** Trial assessments at baseline and 8-week follow-up.

Variable type	Measure
Descriptors and potential moderators	Demographics [44], health literacy [45] COVID-19–related experience [46,47], health conditions, or diagnoses
Feasibility/ Adherence to MOTIVACC^a^	Numbers of messages/images/videos opened and completed; weekly EMAs^b^ opened and completed
Social Cognitive Theory– and motivational interviewing–related mechanisms	Expectations (eg, vaccination benefits or consequences of not being vaccinated) [42,48] Ambivalence regarding vaccination [49,50] Behavioral capability (eg, perceived barriers) [48]
Qualitative, open-ended assessment	An in-app participant-initiated assessment—“Share your thoughts with us”—with an open text field to capture the participant’s thoughts and feelings
Primary outcomes	Vaccine hesitancy and confidence [40,41] Intention to receive the vaccines [42,43]
Secondary outcomes	SARS-CoV-2 vaccine receipt

^a^MOTIVACC: mHealth-based motivational interviewing intervention to promote SARS-CoV-2 vaccination.

^b^EMAs: Ecological momentary assessment.

#### Compensation

All clinical trial participants receive one US $30 gift card as compensation for completion of baseline and 8-week follow-up data collection.

### Statistical Analysis

This pilot study was designed to test feasibility, acceptability, and preliminary efficacy; thus, existing recommendations regarding sample sizes for pilot studies were used [30] versus a formal power calculation. Descriptive statistics will be used to assess the feasibility and acceptability of MOTIVACC, including the proportion of individuals who met inclusion criteria, the proportion of eligible participants who agreed to participate, the proportion of prescheduled events (intervention messages and interactive open-ended questions) delivered, viewed, or completed, and attrition rate at 8-week follow-up. The preliminary efficacy of IMI or MOTIVACC vs. SMI in changing participants’ vaccine hesitancy and uptake intention will be evaluated using generalized linear mixed models, controlling for selected covariates such as sex and education level. Secondary outcomes will be examined using log-binomial regression with an intent-to-treat approach (ie, missing=no vaccine receipt) to compare the effect of IMI or MOTIVACC on the dichotomous 1st-dose vaccine receipt outcome at 8 weeks post enrollment. As aforementioned, given the preliminary nature of the RCT, we do not expect to have sufficient power for full hypothesis testing. For missing data, we will consider a multiple imputation approach based on participants’ demographics at baseline to account for potential missing-at-random mechanisms. We also will explore pattern-mixture and selection models to account for potential (and likely) missing-not-at-random mechanisms [51].

### Ethical Considerations

All study procedures were approved by the Oklahoma State University (IRB-22-282-STW) and University of Oklahoma Health Sciences’ institutional review boards (14893). Electronic informed consent was obtained from all study participants. All CAP participants who completed an interview received one US $30 Amazon gift card distributed via phone or e-mail. All clinical trial participants receive one US $30 gift card as compensation for completion of baseline and 8-week follow-up data collection.

## Results

**Figure 1:**
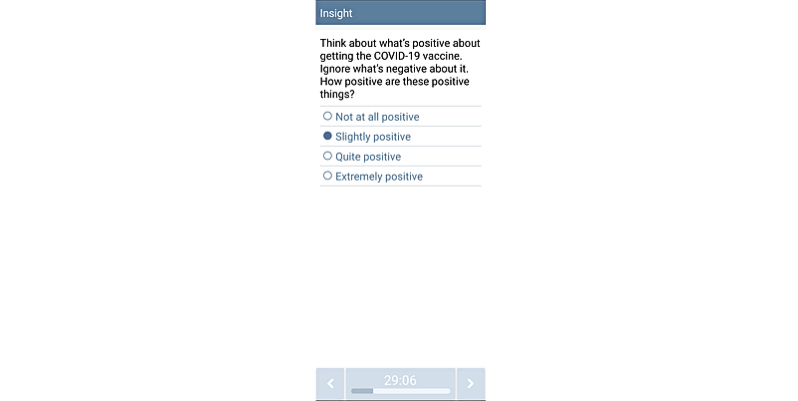
Screenshot of the Insight app.

**Figure 2:**
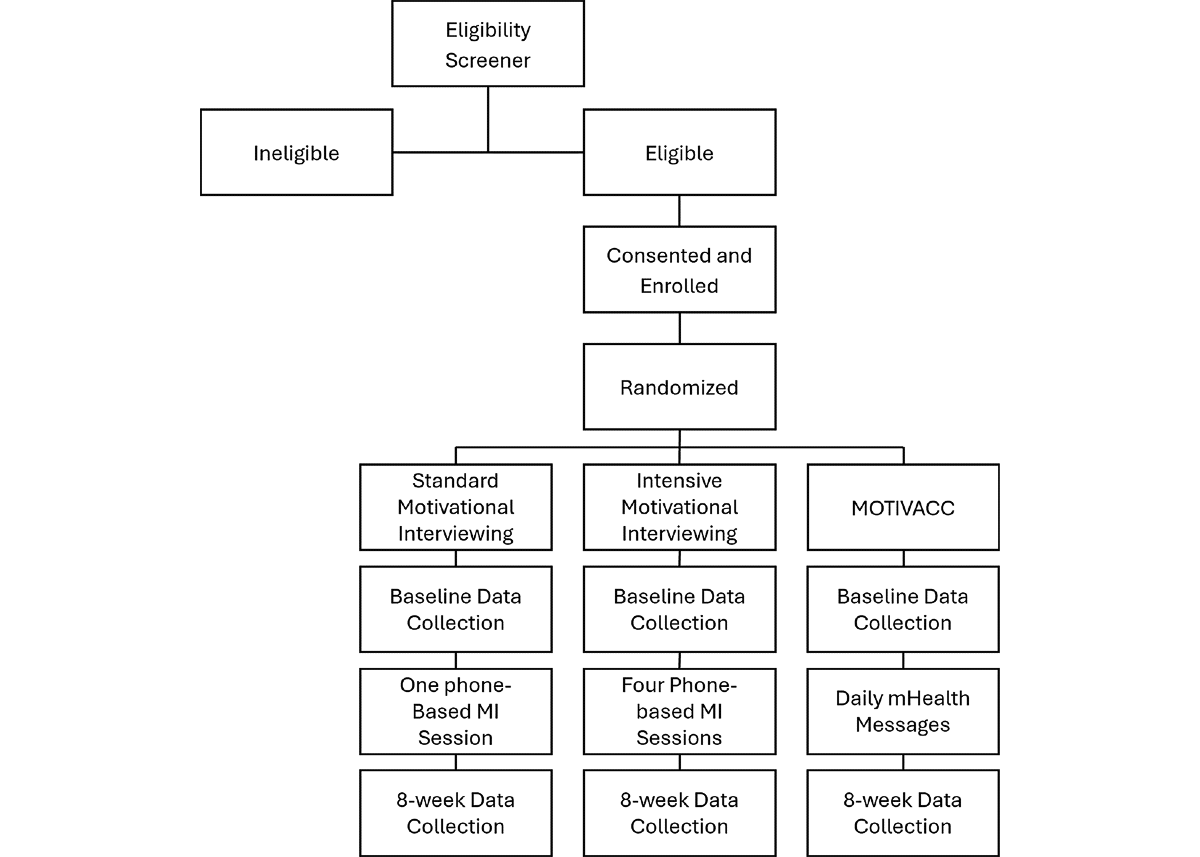
CONSORT (Consolidated Standards of Reporting Trials) flow diagram. MI: motivational interviewing; MOTIVACC: mHealth-based motivational interviewing intervention to promote SARS-CoV-2 vaccination.

This project is funded by the Oklahoma Shared Clinical & Translational Resources Pilot Grant Program, which is funded by the National Institute of General Medical Sciences, a part of the National Institutes of Health (U54GM104938). The study is approved by the institutional review boards of both University of Oklahoma Health Sciences and OSU. As of April 2024, data collection for phase 1 was complete with a total of 16 individuals recruited to participate in the CAP. Qualitative data analysis for phase 1 contributed to the MOTIVACC intervention development and testing (Figure 1). As of December 2024, the planned recruitment goal has been met (ie, n=60, 20/arm) and follow-up assessments are underway. The CONSORT flow diagram is shown in Figure 2, and the SPIRIT checklist is uploaded in Multimedia Appendix 2.

## Discussion

### Anticipated Findings

The results of this study will provide critical data regarding the utility of MI and MI-based mHealth interventions in targeting vaccine-related hesitancy and intention. Despite documented effectiveness, the public health impact of vaccinations is severely limited by misperceptions, hesitancy, and poor acceptance [52]. Messaging from health care providers has not yet been optimized to overcome these barriers [53-55] and has not been tailored to groups that face health disparities, such as rural Americans. Understanding the utility of different communication strategies, including those rooted in empathy, is important to navigate conversations surrounding SARS-CoV-2 vaccines, and others, as alternatives to education or advice. Motivational interviewing is a logical candidate given it is thoroughly operationalized and clearly defined, though questions regarding dose, intensity, and scalability remain. It is essential to investigate the efficacy of MI in promoting vaccine uptake in rural populations to reduce health disparities. Our study will address this need and will contribute to filling the gap in evidence regarding the dose and intensity at which MI can be (cost-) effective. While an app-based mHealth intervention does not allow for two-way or interpersonal communication (a hallmark of MI), careful design of mHealth messages, as well as the ability to share input (eg, “Share your thoughts with us”) could prove sufficient. Conversely, the use of mHealth could help alleviate common concerns that exist in MI-based studies (eg, drift in provider skills), which can be addressed via ongoing evaluation, but this is not overly scalable.

This project will be one of the first to test the use of MI, including via mHealth, to address vaccine hesitancy in adults residing in primarily rural areas. MI has long been acknowledged as a key candidate in helping to navigate difficult conversations, including conversations around general vaccine hesitancy. Although MI’s efficacy has been demonstrated in parents to vaccinate their children, its efficacy among adults, particularly rural ones, to receive vaccines for themselves is unknown. Given the convoluted nature of vaccine hesitancy secondary to the COVID-19 pandemic, autonomy-supportive and empathetic intervention strategies are essential, thus it is critical to evaluate the use of MI in this context.

### Conclusion

Results from this pilot project will be critical for future robust fully-powered clinical trials to examine the efficacy of different MI approaches in promoting vaccination. If MOTIVACC is found to be acceptable and effective, it could be an affordable and scalable stand-alone MI intervention that can be easily and widely implemented with minimal human involvement to reduce the disease in rural populations. The long-term goal of our research is to establish a scalable, automatic, interactive, mHealth-based MI intervention that can be tailored to individuals or groups to decrease vaccine hesitancy for any type of vaccine (not just SARS-CoV-2 vaccine) and increase vaccination rates. Therefore, if successful, MOTIVACC may be adapted to be used in reducing vaccine hesitancy and promoting the uptake of other vaccines. Therefore, this study, together with our subsequent course of research, will help to reduce vaccine-preventable morbidity and mortality in rural populations.

## Appendix

Multimedia Appendix 1 Interview guide.

Multimedia Appendix 2 SPIRIT (Standard Protocol Items: Recommendations for Interventional Trials) checklist.

## Abbreviations

CAP: community advisory panel
CONSORT: Consolidated Standards of Reporting Trials
IMI: intensive motivational interviewing
mHealth: mobile health
MI: motivational interviewing
MOTIVACC: mHealth-based motivational interviewing intervention to promote SARS-CoV-2 vaccination
OSU: Oklahoma State University
RCT: randomized controlled trial
REDCap: Research Electronic Data Capture
SCT: Social Cognitive Theory
SMI: standard motivational interviewing
SPIRIT: Standard Protocol Items: Recommendations for Interventional Trials
WHO: World Health Organization

## Data Availability

Data are deposited in publicly available repositories (where available and appropriate).
