# Supplementary material for: Mobile Health–Based Motivational Interviewing to Promote SARS-CoV-2 Vaccination in Rural Adults: Protocol for a Pilot Randomized Controlled Trial
Source: JMIR Res Protoc. 2025 Apr 28;14:e64010. doi: 10.2196/64010 (PMC12070004; doi:10.2196/64010)
Supplement: Multimedia Appendix 1 [file resprot_v14i1e64010_app1.docx]

**INTERVIEW GUIDE**

| **No.** | **Questions** | **Coding Categories** |
| --- | --- | --- |
| Q1 | Interview identification number (ID)  *[fill out the ID here and in ALL pages of field notes]* | *Type here:* |
| Q2 | Date of data collection | *Month:*  *Day:*  *Year:* |
| Q3 | Name of interviewer(s) | *Type here:* |
| Q4 | Time interview starts | *Hour:*  *Minute:* |

*[Interviewer briefly introduces themselves]*

*[Interviewer/research staff obtains informed consent]:*

First, I’d like to thank you for your participation in our study. We have a few reminders. One, we are interested in your opinion because you have been identified as an important person with relevant opinions in your community. Two, with your permission, we will be recording this conversation today, after which it will be transcribed into text. Your name or any other information that can identify you will be taken out. Three, we will be asking you questions about two general topics: vaccines and the use of mobile health, or, as we’ll call it, mHealth. Do you have any questions?

Great! Okay, before we get started, do we have your permission to record this conversation? IF NO, DO NOT CONTINUE CONVERSATION.

*[Interviewer reminds participant that the interview will be recorded so that participant’s responses can be documented better. Interviewer asks if the participant has any questions about the recording; if not, turns on the Zoom recording and/or the digital recorder]*

*[Interviewer reads aloud all of the information in the table above, so that it can be recorded]*

*[Remind the participant that during the interview, they should not use their real name and should use “I” instead when referring to themselves. Also, do not mention real names of anyone else during the interview. If they want to refer to someone, they can use an initial or a fake name.]*

**INTRODUCTORY QUESTIONS**

Can you please tell us about your job title, position, and responsibility?

Can you tell us a little bit about the institution/organization you work for?

*[Probing question]:* Is it an academic institution, non-profit organization, or private hospital? Is it a general hospital or a clinic/specialist center?

*[Probing question]:* Tell me about your organization’s mission

How many percent of your beneficiaries (i.e., those people who receive your care services) are from rural versus urban areas? An estimate is okay.

Please describe your relationship with members of the community

**MAIN QUESTIONS**

Our first questions are going to be about vaccines. We want to let you know before we start chatting that we respect your opinion, we’re not here to try to change your mind on anything, and there are no right or wrong answers. Sound okay?

First, as you know, we’re interested in discussing vaccines for COVID-19. Can we start with you just sharing your general experiences with COVID-19. Feel free to start back at the very beginning of the pandemic (meaning, March of 2020)

*[Probing question]:* Do people in your community view vaccines positively? Why or why not?

Please tell us about the general impressions of vaccines in your community.

*[Probing question]:* Do people in your community view vaccines positively? Why or why not?

*[Probing question]:* What do people in your community tell you about vaccines?

It will help us better tailor our questions if you share with us whether or not you have been vaccinated for COVID-19?

*[If no]:* Share with me what you don’t like about vaccines, or more specifically, the COVID vaccine.

*[If no]:* Has anyone, like a doctor, nurse, family member, or friend, tried to convince you to get the vaccine?

*[Probing question]:* What was that like?

*[Probing question]:* Did the interaction make you more or less likely to get the vaccine? Why?

*[If no]:* Imagine you were to get the COVID vaccine, what would those circumstances look like?

*[If no]:* What do you think would motivate people like you to get the vaccine?

*[If yes]:* Tell me about your decision to get the vaccine

*[If yes]:* Did anyone, like a doctor, nurse, family member, or friend, tried to convince you to get the vaccine?

*[Probing question]:* What were those conversations like?

*[Probing question]:* Were their attempts effective and why?

*[If yes]:* What, ultimately, made you decide to get the vaccine?

*[If yes]:* What do you think would motivate other people who have not been vaccinated yet to get the vaccine?

- ***If you sense that the individuals’ opinions are inconsistent with those in their community:***

- ***Respondent is vaccinated, but community is largely anti-vaccine*:**

So, am I right in that you opted to get the vaccine even though most in your community have decided not to?

*[Probing question]:* What has that decision been like for you?

- ***Respondent is not vaccinated, but community is largely pro-vaccine:***

So, is it right that you opted not to get the vaccine even though most in your community have decided to?

*[Probing question]:* What has that decision been like for you?

- ***If you sense that the individuals’ opinions are consistent with those in their community:***
- ***Respondent and community are largely anti-vaccine:***

It sounds as though most people in your community are not in favor of the COVID-19 vaccine, is that right?

*[Probing question]:* Why and how do you think that happened?

- ***Respondent and community are largely pro-vaccine:***

It sounds as though most people in your community are in favor of the COVID-19 vaccine, is that right?

*[Probing question]:* Why and how do you think that happened?

Our next questions are going to be about mobile health – or, mHealth. If you’re not familiar with mHealth, it is the use of mobile wireless technologies, like mobile phones, for health. Or in other words, programs delivered via your cell phone to help you with your health.

Have you ever used mHealth or any cell phone app to help you with anything related to your health?

*[If yes]:* Why?

*[If yes]:* What was that like?

*[If yes]:* What did you like about it?

*[If yes]:* What did you dislike about it?

*[If no]:* Why not?

*[If no]:* If you were to use an app for your health, what would be helpful?

*[If no]:* What would not be helpful?

*[If no]:* What about the app would prevent you from using it?

Do you use cell phone apps for anything else outside of your health?

*[If yes]:* How would you describe the apps you like or that are helpful?

*[If no]:* What’s kept you from doing this?

Think more broadly about your community. How would members of the community perceive mHealth apps?

*[Probing question]:* What would make them less or more likely to use them?

**CLOSING QUESTIONS**

Do you have any other comments?

Do you have any questions for us related to this study?

*[Explain to the participant that the following questions are for data analysis purposes.]*

How old are you?

What is your gender?

In what county do you work?

*[Thank the participant.]*

*[Read aloud so that it is recorded]:* This is the end of the interview. Time: HH-MM *[Turn off the voice recorder]*

| **No.** | **Questions** | **Coding Categories** |
| --- | --- | --- |
| Q5 | Time interview starts | *Hour:*  *Minute:* |
